# Integrating cultural dimensions in sperm whale (*Physeter macrocephalus*) conservation: threats, challenges and solutions

**DOI:** 10.1098/rstb.2024.0142

**Published:** 2025-05-01

**Authors:** Ana Eguiguren, Isabel Avila, Sarah Mesnick, Mauricio Cantor, Taylor Hersh, Héctor Pérez-Puig, Patricia Rosero, Luke Rendell, Hal Whitehead, Constanza Rojas, Juan Jose Alava

**Affiliations:** ^1^ Department of Biology, Dalhousie University, Halifax, Nova Scotia, Canada; ^2^ Institute for Terrestrial and Aquatic Wildlife Research (ITAW), University of Veterinary Medicine Hannover, Hannover, Niedersachsen, Germany; ^3^ Grupo de Investigación en Ecología Animal, Universidad del Valle, Cali, Colombia; ^4^ Marine Mammal and Turtle Division, Southwest Fisheries Science Center, National Marine Fisheries Service, National Oceanic and Atmospheric Administration, La Jolla, CA, USA; ^5^ Department of Fisheries, Wildlife and Conservation Sciences, Oregon State University, Newport, OR, USA; ^6^ Marine Mammal Institute, Oregon State University, Newport, OR, USA; ^7^ Marine Mammal Program, Prescott College Kino Bay Center for Cultural and Ecological Studies, Kino, Bahía de Kino, Mexico; ^8^ Escuela de Ciencias Ambientales, Universidad de Especialidades Espíritu Santo, Samborondon, Guayas, Ecuador; ^9^ School of Biology, University of St Andrews, St Andrews, Fife, UK; ^10^ Centro de Estudios Avanzados en Zonas Aridas, La Serena, Coquimbo, Chile; ^11^ Ocean Pollution Research Unit, Institute for the Oceans and Fisheries, University of British Columbia, Vancouver, British Columbia, Canada

**Keywords:** Eastern Tropical Pacific, behaviour, conservation, anthropogenic threats, social transmission, sperm whale clans

## Abstract

Culture—socially transmitted behaviours shared within a community—can influence animal populations' structure, vulnerability and resilience. Clans of sperm whales in the Eastern Tropical Pacific (ETP) exemplify the profound influence of culture on these dynamics and highlight the challenges of accounting for culture in conservation efforts. Globally, sperm whales are classified as vulnerable, and the ETP sperm whale population has struggled to reach a positive growth rate. This stagnation is partly due to cumulative anthropogenic threats in the region, including fishing conflicts, vessel traffic, pollution, deep sea mining, oil and gas exploration, and anthropogenic climate change. The United Nations Convention on Migratory Species adopted a Concerted Action for ETP sperm whales in 2017, proposing collaborative efforts to address cultural dimensions in conservation. However, knowledge gaps and real-world implementation challenges persist. Here, we review the role of social transmission in shaping sperm whale behaviour and populations, outline current anthropogenic threats and environmental stressors they face in the ETP, and discuss the ongoing challenges of incorporating cultural dimensions into large-scale international conservation efforts. Strengthening transnational collaboration and capitalizing on new technologies for efficient analysis can help bridge these knowledge gaps and enhance future research on this iconic species.

This article is part of the theme issue ‘Animal culture: conservation in a changing world’.

## 1. Introduction

The effect that socially transmitted behaviours can have on the ability of a population to persist over time is profound [1–3]. Researchers increasingly recognize the importance of accounting for culture—defined as socially transmitted behaviours shared within a community—to more accurately assess population structure, vulnerability and resilience of species whose behaviours are moulded through social transmission [4]. The role that culture can play in informing conservation efforts was highlighted early on in cetacean species with substantial evidence of cultural transmission [5,6]. Among these, sperm whales (*Physeter macrocephalus*) from the Eastern Tropical Pacific (ETP) ocean have been a flagship species illustrating how deeply culture can affect population structure, behavioural flexibility and vulnerability to changes [2,5,7].

Sperm whales are globally classified as vulnerable by the International Union for Conservation of Nature (IUCN), as their global population size remains below 50% of inferred levels three generations ago as a result of intensive 20th-century whaling [8]. Despite the end of commercial whaling following the International Whaling Commission’s 1986 moratorium, population growth worldwide has stagnated, with limited cases of slow recovery and some instances of concerning decline [9]. This is partly due to sperm whales’ slow life history and low reproductive rates, with females producing one calf every 4−6 years [9,10]. Additionally, sperm whales currently face contemporary regional and global threats to their marginal capacity for recovery [10]. Notably, populations in human-impacted areas, such as the Mediterranean and the Caribbean Seas, have declined severely due to fishing gear entanglements, vessel traffic and unmanaged tourism [11,12].

Following Resolution 11.23 on the Conservation Implications of Cetacean Culture [6], the Convention on the Conservation of Migratory Species of Wild Animals (CMS) of the United Nations Environment Programme adopted the Concerted Action for Sperm Whales of the ETP. This collective effort relies on evidence for the treatment of sperm whale cultural clans as ‘socially significant units’. It proposes the creation of a collaborative research network for CMS member states and beyond (Mexico, Costa Rica, Panama, Colombia, Ecuador, Peru and Chile) [13]. This effort aims to clarify how culture structures ETP sperm whale populations and determine whether clans could be treated as independent conservation units. However, sperm whales in the ETP are highly nomadic, roaming over vast home ranges of thousands of kilometres, making monitoring challenging [14]. Moreover, the ETP faces increasing human activities and unpredictable oceanographic changes that can impact sperm whales’ well-being [15,16]. However, as highlighted by subsequent reports submitted to CMS [17], while culture likely influences sperm whale population structure, vulnerability and resilience to anthropogenic threats, there remain significant knowledge gaps and barriers to incorporating a cultural dimension into the conservation of the species.

Our goals here are fivefold. We (i) review current knowledge on the role of social transmission in propagating sperm whale behaviours in the ETP, (ii) detail anthropogenic threats faced by sperm whales in this region, and (iii) describe how this knowledge can inform conservation efforts. We then (iv) discuss the challenges of incorporating culture into the existing conservation framework for sperm whales. We conclude by (v) providing recommendations to overcome these obstacles and highlighting key research avenues for the future.

## 2. Sperm whale culture in the Eastern Tropical Pacific ocean

### (a) Sperm whale culture

**Figure 1.**
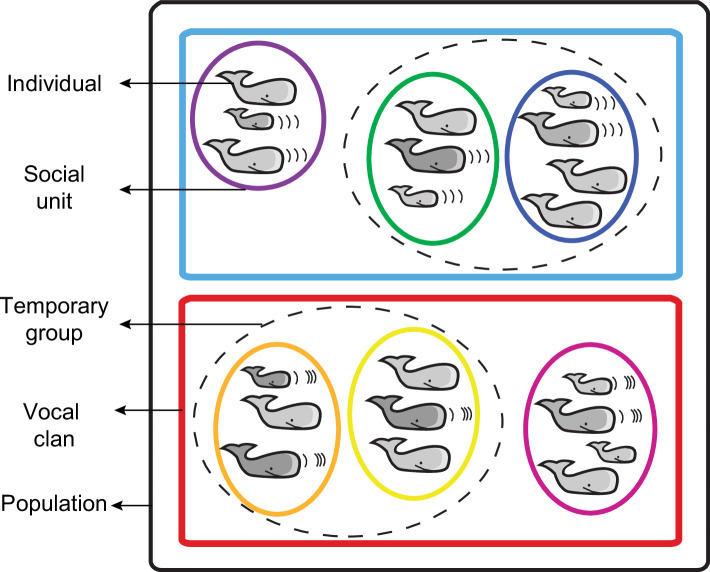
Multi-level social structure of sperm whales. Females and juvenile whales form nearly permanent social units (coloured ellipses) that can create temporary groups (dashed ellipses), usually with social units with which they share a significant portion of their coda repertoires, forming vocal clans (coloured rectangles) within the same geographic region (black rectangle). Schematic adapted from Cantor *et al*. [20].

Social transmission largely shapes sperm whale behaviour, movements and population structure. While mature males—who traverse the ocean from tropical to sub-polar waters—are fairly solitary, females and juveniles—mostly restricted within tropical and temperate waters—are highly social and have a complex, multi-level social structure [9,18,19] (figure 1). At the lowest level, individuals within a *social unit* hold stable and long-lasting social bonds with an average of 11 other individuals, some matrilineally related [21–23]. Individuals from different social units associate temporarily (hours–days), forming a *social group* [24]. However, individuals of different social units only interact with units belonging to the same cultural *clan*, representing the highest social level. Clans are identifiable to researchers based on similarities in the repertoire of their social vocalizations, referred to as *codas* [25]. Codas consist of 3−20 temporally patterned clicks produced by sperm whales in social contexts, often exchanged between individuals [26]. Codas are algorithmically classified into types based on the number and temporal pattern of clicks [27,28]. Thus, clans are collections of social units that frequently use the same coda types [20]. Sperm whales from different clans can be found in sympatry in a region (i.e. <50 km apart) while still maintaining social isolation [20]. Clans can include thousands of individuals across thousands of kilometres within an ocean basin, many of which may never come in contact with each other [29].

Beyond distinct vocal repertoires, sperm whales from different clans have distinct movement patterns, distributions, and fitness, foraging, parental and social behaviours (electronic supplementary material, table S1). These behavioural differences have been identified among sympatric clans, suggesting that these differences are not the product of environmental drivers [25,30]. Instead, the behavioural variations among clans most likely emerged through social transmission—by learning from preferred associates—and became fixed via social conformity—a tendency to imitate behaviours prevalent in one’s social group [31].

### (b) Sperm whale clans in the Eastern Tropical Pacific

A recent analysis found seven clans in ETP waters (*Four-Plus*, *Palindrome*, *Plus-One*, *Regular*, *Rapid Decreasing*, *Rapid Increasing* and *Short*) [32]. While some clans have been detected within a narrow range in the ETP (e.g. *Plus-One* found only off the Galápagos Islands and mainland Ecuador), others (e.g. *Short*) span the entire Pacific Ocean basin [33]. Although this work provides a basin-wide panorama of clan distributions, long-term surveys off the Galápagos Islands showed that clan distribution in the ETP can shift over time. From 1985 to 2014, sighting rates of sperm whales in the region shifted from relatively abundant in the late 1980s and early 1990s to non-existent in the 2000s, followed by a slight increase after 2010 [34]. Remarkably, clans that were frequent in the 1980s and 1990s (*Regular*, *Plus-One*) were entirely replaced in the 2010s (by the *Four-Plus* and *Short* clans) [35], likely due to movements between the waters off the Galápagos Islands and the broader ETP [34,35].

## 3. Threats to sperm whales in the Eastern Tropical Pacific

**Figure 2.**
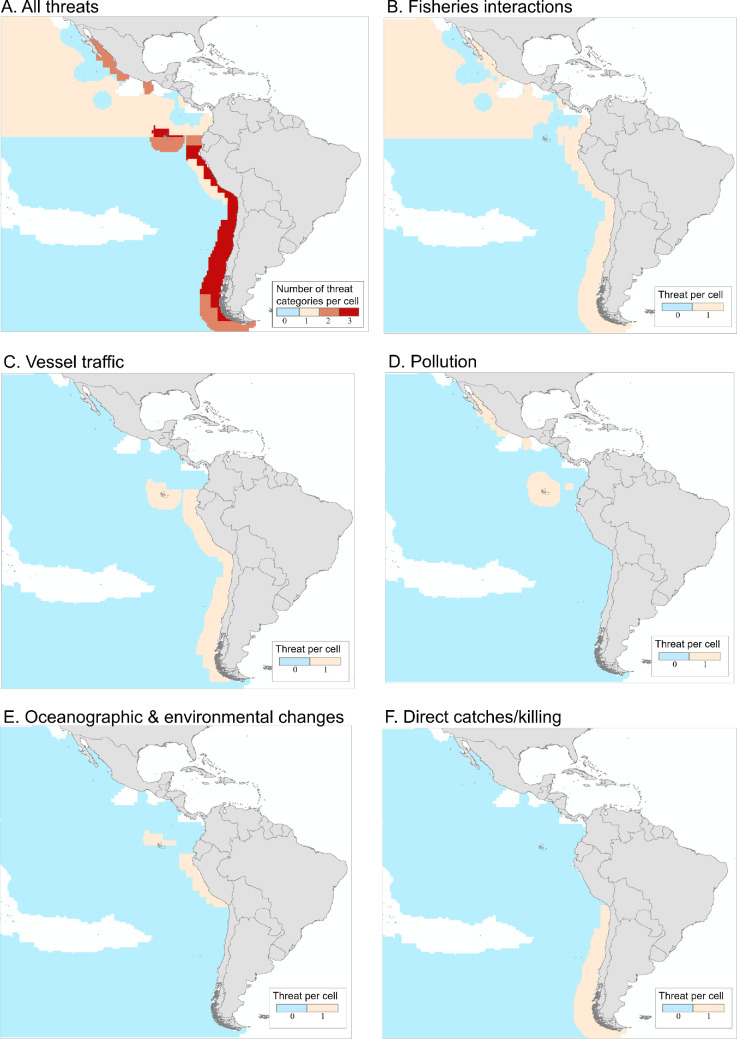
Risk maps for sperm whales in the ETP. (A) Cumulative risk estimated as the intersection between threats documented between 1991 and 2024 and the modelled core habitat of the species. Red cells show areas of high risk where at least three threats were detected per cell (*N* threats = 5; AquaMaps presence probability threshold ≥ 0.6). The blue region represents the core sperm whale habitat where no threats have been documented. (B–F) Threat-specific risk maps for (B) fisheries interactions, (C) vessel traffic, (D) pollution, (E) oceanographic and environmental changes and (F) direct kills. Tan cells show where each threat has been documented, and blue cells show the core sperm whale habitat, where a specific threat was not documented. Adapted and updated from Avila *et al.* [36].

In the ETP, sperm whales face multiple co-occurring regional and global human-induced threats that may have compounded effects on their health and survival (figure 2) [8,18,37].

### (a) Global climate change

**Figure 3.**
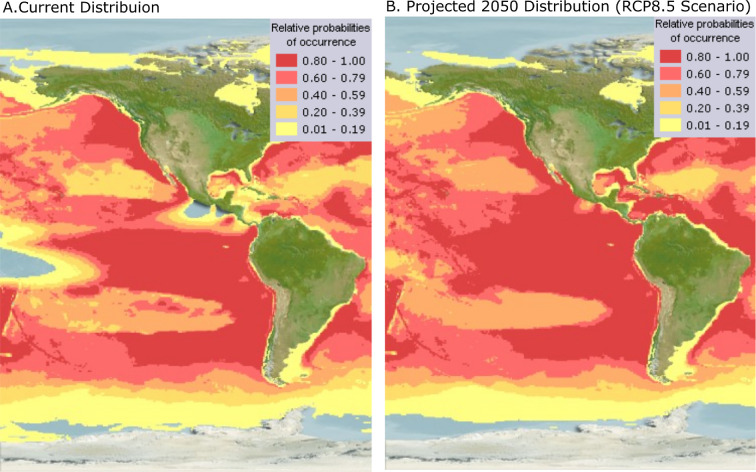
Distribution map of Eastern Pacific sperm whales. (A) Current sperm whale native home range distribution map. (B) Projected sperm whale native home range distribution map for 2050 under the RCP8.5 climate change scenario. The RCP8.5 is a representative concentration pathway (RCP) where greenhouse gas emissions continue escalating to the highest concentration in the absence of effective climate change policies, resulting in a 4.3°C temperature rise by 2100 [39,40]. The scale bar shows the relative probability of occurrence of sperm whales, ranging from 0.01−0.19 (i.e. light yellow) to 0.80−1.0 (i.e. red), standing for 1.0−20% and 80−100% probability of occurrence. Maps were computer generated and adapted from AquaMaps (2019) online (i.e. for *P. macrocephalus* (https://www.sealifebase.ca/summary/Physeter-macrocephalus.html) with modelled year 2050 native range map based on IPCC RCP8.5 emissions scenario; https://www.aquamaps.org).

Globally, climate change will likely affect sperm whale foraging success, behaviour and distribution [38]. In the ETP, the home range of sperm whales is predicted to change considerably under the business-as-usual scenario, with increasing probabilities of occurrence in higher latitudes [39,40] (figure 3). In the Northeast and Central Pacific, modelled sperm whale occurrence probabilities are expected to rise from 1–39% to 40–59%. This shift would likely reflect distribution and biomass changes of sperm whales’ deep-dwelling cephalopod prey, which are highly susceptible to oceanographic shifts [41–43]. Previously, anomalous warming in the ETP has resulted in the collapse of the Humboldt squid (*Dosidicus gigas*, a main prey item for sperm whales [41,44]), an expansion of the Humboldt squid’s distribution [33,45], declines in sperm whale feeding success [37] and large-scale movements of sperm whales from important aggregation zones [46].

### (b) Fisheries interactions

Locally, sperm whales could be threatened by interactions with fisheries. Most documented sperm whale deaths in the region have been associated with fisheries interactions [36,47,48] (figure 2B), primarily entanglements with gillnets and longlines [49–51]. Although reports are scarce along ETP coastlines (*ca* 104 reports made between the 1980s and 2020), these numbers should be interpreted cautiously [49,50,52–54]. An absence of strandings may indicate movements of sperm whales away from continental waters [50], as well as causes of death associated with a higher probability that a whale will sink far offshore.

Off southern Chile, as in other regions, sperm whales frequently depredate Patagonian toothfish (*Dissostichus eleginoides*) longlines, which has led to entanglement and even a gunshot as an aversive measure by a fisherman [55–57]. Still, the impact of this interaction on local sperm whale well-being remains unclear (figure 2F). These interactions likely involve mature/maturing males, as they have only been documented in cold temperate waters. Though these interactions take place outside the ETP, their impact on male survival could have cascading effects on ETP sperm whale populations if these males would have otherwise reproduced with ETP females [58].

Moreover, a fishing fleet that targets Humboldt squid along the Humboldt Current has increased exponentially in recent years. This international fishery frequently operates in seas outside national jurisdictions. In past decades, catch in the fisheries along the Humboldt Current has increased from 12 550 tonnes in 1990 to 816 914 in 2019 [59,60]. Past work suggests human consumption of squid does not deplete the resources for squid-eating marine mammals [61]. However, the expanding fleet, its overlap with sperm whale distribution, updated sperm whale population estimates and changing oceanography warrant a reassessment of this assumption in the region. For instance, in the Gulf of California, the collapse of the Humboldt squid fishery—likely resulting from overexploitation and changing oceanographic conditions—likely explains the disappearance of sperm whales in the region since 2015 [46].

### (c) Vessel collisions

Vessel collisions are a known cause of sperm whale mortality worldwide [62,63]. However, limited data on both their frequency and potential contributing factors impede any reliable assessment of prevalence and trends throughout the ETP. Evidence suggests vessel collisions with sperm whales may be more common than previously suspected in zones with intense traffic (e.g. Canary Islands and Mediterranean Sea), posing a significant threat for sperm whales in these regions [64,65]. For example, in the Midriff Islands Region in the Gulf of California—home to an estimated 350 sperm whales—intense industrial and artisanal vessel traffic complicates efforts to reduce collision-related sperm whale deaths. Throughout the ETP, collision risk is likely to increase with the development of new large-ship routes and commercial ports.

### (d) Oceanic contamination

Oceanic contamination also threatens individual sperm whale health and survival within the ETP [15,66] (figure 2D). Biomarker analyses of skin samples from the Pacific Ocean showed that whales off the Galápagos Islands had the highest levels of enzymes associated with organic compound contamination (e.g. polycyclic aromatic hydrocarbons (PAHs) and persistent organochlorine pollutants (POPs)) [66,67]. Microplastics have been detected in all water and organism samples across the ETP [68], including in zooplankton and fish around the Galápagos Islands [69,70]. Concerningly, micro- and macro-plastics were found in most Humboldt squid tissues off Ecuador and the Humboldt Current [71,72]. In other regions of the world, the death of stranded sperm whales has been linked to the individual consumption of several tons of plastic, often discarded fishing gear [73–75].

### (e) Acoustic pollution

Acoustic pollution—generated by naval operations, seismic exploration associated with offshore oil and mineral extraction, sonar and vessel traffic [76]—can travel hundreds of kilometres and can impair sperm whales’ ability to echolocate, displace them from their habitats, damage their auditory organs and even cause death [61]. Vessel traffic and its associated noise in the ETP is concentrated near coastal waters, particularly off northern Peru, Ecuador, Panama and the southwest and west coasts of Mexico, where it could pose a significant threat to sperm whales [77]. Although there are no reports on sperm whale deaths, injuries or behavioural responses attributed to acoustic pollution in the ETP, this does not eliminate the possibility that sperm whales are affected by this threat.

### (f) Deep-sea mining

Deep-sea mining activities can also impact ecologically important species and populations, including sperm whales’ squid prey, through habitat destruction and the production and resuspension of large, persistent sediment plumes, metals and toxic organic pollutants [69,78,79]. While marine mining is limited in the ETP, there are signs that member states aim to expand their extractive frontiers toward the oceans. For example, the Clarion–Clipperton Zone is a large region in the Pacific Ocean between Hawaii and Mexico for which use contracts have been granted by the International Seabed Authority [80].

### (g) Geographic range of threats

Most threats have been documented in nearshore waters, where human populations and activities are concentrated, particularly off Ecuador, Peru and Chile (figure 2). Here, sperm whales face significant risks from fisheries interactions, especially in oceanic and nearshore waters off mainland Ecuador [81–83], where major industrial and small-scale (artisanal) fishing fleets operate [84,85]. Notably, high levels of risk to sperm whales have been registered around the Galápagos Islands despite it being a marine protected area (MPA), particularly associated with high levels of illegal, unreported and unregulated (IUU) fishing from Asian nations in and around the MPA boundaries [86,87] (figure 2). However, the absence of risks shown in figure 2 does not necessarily mean no threats exist in a region. Instead, this most likely reflects the limited information on threats available for some areas [36,88].

## 4. The implications of social learning for sperm whale conservation

### (a) Social learning and adaptability

The ability of sperm whales to socially learn has important implications for the speed with which they can adapt to change, which may have dual consequences for their survival. On the one hand, the ability to adopt behaviours in a short period could confer sperm whales resilience against novel threats [2]. For example, the early decline in harpooning success rates during open-boat whaling in the 18th and 19th centuries likely resulted from the rapid social transmission of defensive strategies among groups of sperm whales [89]. However, social transmission can also spread behaviours that create conflicts. For instance, the social diffusion of depredation on Alaskan demersal fishing lines among mature male sperm whales has increased conflicts with fishers in the region [90].

Conversely, if sperm whales favour ‘traditional’ over novel behaviours, they may be unable to adapt to changing conditions [91]. This is exemplified by southern resident killer whales (*Orcinus orca*), whose culturally transmitted preference for Chinook salmon (*Oncorhynchus tshawytscha*) has resulted in significant southern resident population declines as Chinook salmon availability has decreased despite viable alternative prey options [92]. Understanding the dynamics by which sperm whale behaviour can (or cannot) adapt to changing conditions will improve our ability to predict the response and trajectory of sperm whale populations to anthropogenic threats.

### (b) Key individuals as repositories of knowledge

In long-lived social species, older individuals can hold ecological knowledge crucial to the survival of their group [93,94]. Among sperm whales, older females may significantly contribute to their social unit’s fitness despite their depleted reproductive capacity [9]. The value of key individuals as knowledge repositories in sperm whales can be illustrated by the prolonged effect of industrial whaling on sperm whale populations in the ETP, even after its cessation [26,95]. A current hypothesis suggests that removing a vast proportion of the sperm whales’ population not only eliminated individuals but also valuable ecological knowledge, contributing to the low reproductive rates for 15 years after the whaling moratorium [58]. Population viability assessments need to account for the disproportionate contribution of older, non-reproductive females to a group’s fitness to accurately represent a population’s recovery potential.

### (c) Culturally distinct units

Conservation legislation and organizations (IUCN, US Endangered Species Act, Canada’s Species at Risk Act) generally define sub-species level conservation units as discrete populations/social groups with ecological or conservation significance. Although the requirements to meet these criteria are debated [96–98], recent efforts have incorporated culturally transmitted behavioural variability into delineating conservation units and informing management [99,100]. Delineating conservation units based on cultural distinctions can improve management outputs if these are stable, heritable and impact demography, fitness and conservation outputs [4,99,100]. Moreover, data on socially transmitted acoustic repertoires are usually easier to collect than other traditional lines of evidence (e.g. genetics or morphology), which may enable us to elucidate population structure and conservation priorities more efficiently [101].

The distinct dialects of ETP sperm whale clans reflect social segregation maintained across generations, reflected by differences in maternally inherited mitochondrial haplotypes between the clans [102]. Evidence suggests that sperm whale dialects in the ETP have evolved to increase clan identifiability, particularly among sympatric clans [32], which highlights the importance of distinct dialects to the whales themselves. Thus, ETP sperm whale clans represent a stable level of population structure holding inherent conservation value [100].

Additionally, some culturally transmitted behavioural differences among sperm whale clans in the region may be associated with different levels of vulnerability to anthropogenic threats and oceanographic changes. ETP sperm whale clans have been found to respond differently to anomalously warm temperatures during El Niño–Southern Oscillation events, suggesting some may be more intrinsically vulnerable to extinction (electronic supplementary material, table S1) [103,104]. Similarly, like populations with narrow home ranges, clans with a narrow geographic distribution may be more intrinsically vulnerable to extinction than more widely distributed clans [32]. However, the stability of these behavioural differences over time and their relevance for directed conservation remains uncertain.

## 5. Ongoing challenges to the effective conservation of Eastern Tropical Pacific sperm whales

### (a) Knowledge gaps

Adequate conservation relies on defining conservation units and assessing the severity of threats to their survival. This is difficult to achieve at relevant temporal and spatial scales for sperm whales due to their deep-diving behaviours, vast oceanic home ranges, nomadic movements and long life histories. Namely, long-term and wide-ranging monitoring are prohibitively costly to research groups in nations along ETP coasts [7]. Most research on sperm whales in the region has been conducted and financed by groups from high-income countries (e.g. Canada, USA and UK), while local research is often opportunistic or self-funded. This has resulted in significant gaps in our understanding of sperm whale population structure, movements, exposure and vulnerability.

Current status assessments and population models refer to populations delineated by ocean basins or stock designations based on geographic boundaries and—sometimes—movement and genetic data [105–107]. Moreover, direct measurements of human impacts on sperm whales’ individual- and population-level well-being are extremely rare. Consequently, our understanding of the vulnerability of sperm whales in the ETP to threats is mainly extrapolated from studies elsewhere (e.g. the Mediterranean [11]), based on a few stranding reports [47,48,108], inferred from population trajectory models, or gleaned from models of sperm whale habitat and threat overlap [95,109].

While incorporating a cultural dimension could improve assessments and policies directed at sperm whale conservation in the region, available data are currently insufficient. Research on ETP sperm whale clan behaviour and ecology stems from surveys conducted between 1985 and 2014 off the Galápagos Islands and, sporadically, the west coast of South America (electronic supplementary material, table S1). But, despite this decades-long effort, our understanding of sperm whale clans’ behaviour, movements and connectivity represents a fragment of sperm whales’ distribution and lifespan, limiting our capacity to assess their vulnerability at a clan level.

Additionally, research in the region has almost entirely focused on females and juvenile sperm whales, leaving mature males out of the picture. Sperm whales are extremely sexually dimorphic [26]. Unlike females, whose movements are restricted within ocean basins, males can travel and reproduce across basins and with multiple clans [110]. The morphological differences between male and female sperm whales seem to result in distinct ecological niches [111–113]. Mature males are, therefore, exposed to distinct threats—such as interactions with demersal fisheries in circumpolar waters [55,114]—and may have different responses to oceanographic changes than females. Currently, we do not understand the natal origins, movements or vulnerability of mature males that mate and forage in the ETP, nor how clan identity influences mating behaviours [115]. While some mature males encountered in the Gulf of Alaska have natal ties with the Gulf of California, it is uncertain whether this link extends further south [106]. Still, declines in the adult male population can severely affect overall reproductive rates many years after the causes of mortality are eliminated [58,95].

### (b) Existing conservation instruments and legislation

Conservation instruments and legislation that apply to ETP sperm whales do so either at a species or stock level (electronic supplementary material, table S2). Stock boundaries for sperm whales often reflect geographical and national boundaries [105] and may incorporate movement and genetic data [106,107]. However, these definitions fail to capture the species’ social and sexual demographic structure.

Some individual nations’ legislation may provide avenues to include a cultural dimension to sperm whale conservation. For example, the US’s Endangered Species Act [116] contemplates protection for Distinct Population Segments, defined as discrete and ecologically significant population segments that warrant threatened conservation status. Sperm whale clans likely fit the criteria for discreteness and significance [100], although it is unclear if clans are differentially threatened. Recent guidelines for stock assessment reports for the US’s Marine Mammal Protection Act also acknowledge that conservation units can be delineated based on movements, distribution and acoustic call types [117], which are distinct among sperm whale clans. Likewise, Ecuador’s Constitution recognizes and grants rights to ‘nature or Pacha Mama’, including to the preservation of its ‘vital cycles, structure, functions and evolutionary processes’, which could encompass sperm whales’ culturally transmitted behaviours. However, not all ETP nations provide the legal framework for the management and conservation of culturally distinct units.

Finally, the protection of sperm whales and other cetaceans in the region is achieved through MPAs, which, despite restricting threatening human activities, are unable to protect highly mobile species fully [118–120]. Moreover, fixed geographic areas are insufficient to protect highly mobile species like sperm whales, whose home ranges extend beyond MPAs and national jurisdictions, where monitoring and enforcement responsibilities are unclear.

## 6. Ways forward

The threats and challenges to conservation faced by sperm whales echo those experienced by other long-lived, highly mobile oceanic species, including other cetaceans, whose behaviours are largely shaped by social learning [121–124]. Moreover, when these species’ home ranges span waters outside nations’ jurisdiction, similar logistical, economic and political challenges arise [125]. The following actions, while focused on ETP sperm whales, apply to other species and regions sharing similar issues.

### (a) Strengthen trans-national collaboration to support large-scale, long-term research

Collaboration in data collection, sharing and analyses among researchers from nations traversed by sperm whales is essential to inform conservation efforts at appropriate temporal and spatial scales. Establishing the ‘Cachalotes del Pacifico’ network in 2022, which includes researchers from 17 countries, was a key step towards facilitating collaboration. However, the report on the implementation of the 2017 Concerted Action [13] highlights a lack of funding for research groups based in ETP nations. Recognizing that several of these nations are emerging economies faced with profound social struggles and political instability, the support of institutions in high-income countries will be crucial in filling existing knowledge gaps and bolstering effective conservation. Effective and sustainable collaboration will require that resources support local research and conservation capacities and that locally informed conservation needs are prioritized [126].

For instance, stranding reports are an extremely valuable and low-cost source of information on sperm whale and other cetacean species’ distribution and the threats they face [47,50,83]. Some progress has been made in Mexico; the Mexican Marine Mammal Stranding Network, composed of multiple non-government organizations, currently monitors over 10 000 km of the nation’s coastline. However, other ETP nations have struggled to sustain the equipment and logistics required for maintaining stranding networks despite repeated initiatives [127,128]. As a result, stranded individuals are disposed of before data collection or reporting. In Ecuador, no stranded sperm whales have undergone a complete necropsy to confirm the cause of death since 1987, making it impossible to differentiate anthropogenic from natural causes of death (e.g. interspecies interactions, disease and environmental stress). It is therefore essential that collaborative efforts prioritize funding local stranding networks and support the capacity building of local specialists to contribute to our understanding of the threats faced by sperm whales and other cetaceans in the region while ensuring the safety of involved participants.

Open and equitable access to data, research tools and publications is key to facilitating collaboration. Recently developed open-access photo-identification software, like Flukebook and HappyWhale, allows researchers to match their catalogues to those held by colleagues from across the world, which will help reveal individual movements of sperm whales in a cost-effective way [129,130]. Unfortunately, the lack of connection between these platforms has limited progress toward understanding sperm whale movements globally. We should work towards the creation of an ETP-wide sperm whale catalogue that is managed and maintained by colleagues throughout the region. We likewise encourage open-access code and software—like the IDcall algorithm used to determine clan identity, available as an R script [27]—to facilitate the implementation of consistent analyses across research groups. Importantly, ensuring free knowledge accessibility—including providing Spanish translations of research outcomes and software—will be essential to ensuring the adoption of these technologies.

### (b) Incorporate new technologies for efficient analysis

Incorporating new technologies into a collaborative approach can also expand our comprehension of sperm whale movements, distribution and vulnerability in the region. For instance, deep learning neural network algorithms have been incorporated into open-access photo-identification matching software, making matching across large catalogues much more efficient. This can reveal important information about individual movements through the ETP and beyond [129,130]. Moreover, this technology is available for the photo-identification of other cetacean species, which rely on a variety of morphological features for identification and matching.

Strategically placed autonomous hydrophones can record sperm whale vocalizations over prolonged periods (e.g. months to years) regardless of oceanographic conditions. Analyses of such recordings can elucidate the seasonal distribution of sperm whales in a region [131], group sizes [132], demographic structure [133] and clan identity [32]. Recently developed sperm whale echolocation and coda detection software will dramatically reduce the time required to analyse large acoustic datasets [134], making the data analyses more feasible. Similar species-specific automated acoustic detection technology based on open-access software has been developed for other cetacean species [135,136]. Thus, if the setup and deployment of autonomous hydrophones are done in collaboration with experts from other fields, acoustic data collected could serve to inform on the distribution and temporal patterns of multiple cetacean species simultaneously.

Using drone photogrammetry can also provide valuable insights into the health and exposure to threats of sperm whales and other marine megafauna in the region. For example, aerial body condition measurements can be used to indicate an individual’s health [137–139], and aerial photography can also reveal the incidence of human-induced scarring [140]. Comparing these metrics across segments of the sperm whale population (e.g. clans, sexes and regions) could help identify vulnerable groups towards which conservation actions can be directed.

## 7. Concluding remarks

The cultural dynamics of ETP sperm whales significantly influence their population structure and behaviours, underscoring the importance of incorporating cultural dimensions into conservation efforts. Despite the significance of culture for the ETP clans of sperm whales, our understanding of their population dynamics, movements and vulnerability to threats remains limited. This gap is primarily due to the challenges posed by their oceanic habitat, extensive nomadic movements, vast home ranges and long lifespans, compounded by insufficient research and conservation resources in ETP nations. Addressing these challenges requires a multi-faceted approach. First, establishing equitable and inclusive collaborations between ETP nations and high-income countries can enable local research through funding and capacity-building initiatives. Second, adopting new, cost-effective technologies can enhance our understanding of sperm whale movements and behaviours. Finally, prioritizing data sharing and ensuring open access to research findings will advance our understanding of sperm whale biology and promote effective conservation strategies. By bridging these knowledge gaps and fostering international collaboration, we can better safeguard these iconic marine predators and their unique cultural traits, ensuring their survival amidst increasing human-induced threats.

## Data Availability

Supplementary material is available online [141].
